# Nucleolin-Targeted DNA Nanoflowers Enable Multimodal Synergistic Cancer Therapy

**DOI:** 10.34133/bmr.0254

**Published:** 2025-09-23

**Authors:** Anwen Ren, Huan Liu, Zimei Tang, Peng Zheng, Qingyi Hu, Tao Huang

**Affiliations:** Department of Breast and Thyroid Surgery, Union Hospital, Tongji Medical College, Huazhong University of Science and Technology, Wuhan, China.

## Abstract

Copper plays multifunctional roles in both physical processes and cancer development. Since copper is an excellent candidate for Fenton-like reactions and the inducer of cuproptosis, copper-based antitumor drugs have attracted many researchers in recent years. However, there are still some barriers to their clinical application, such as leakage to normal tissues, excess of glutathione (GSH), and lack of H_2_O_2_ in the tumor microenvironment, indicating that copper alone is not enough for cancer therapy. Herein, we constructed a DNA-based nanodrug loaded with Cu^2+^ and glucose oxidase (GOx) for synergistic cancer therapy, namely, glucose oxidase-copper-DNA hybrid nanoflower (GCD). AS1411 aptamer, coded in the long single-stranded DNA sequence, provided GCD with tumor-targeting ability, enhancing its bio-safety. The addition of GOx not only provided adequate H_2_O_2_ but also helped deplete GSH. Besides, as it oxidated glucose to gluconic acid, the main energy source of tumor cells was cut off. The in vitro and in vivo antitumor ability of GCD was verified. We also examined immune cell death induction and the immune regulation role of GCD and found that the combination of anti-programmed death-1 antibody further enhanced its antitumor effect. These results contribute to the further study and application of copper-based drug development.

## Introduction

Copper plays essential roles in numerous biological processes, including mitochondrial respiration, antimicrobial, and immune regulation [1]. In cancers, it participates in several signaling transduction pathways related to tumorigenesis, tumor growth, migration, metabolism, and angiogenesis [2,3]. However, excessive copper can exert antitumor effects through oxidative stress, proteasome inhibition, and antiangiogenesis [4]. Fenton or Fenton-like reactions utilize metal ions and hydrogen peroxide (H_2_O_2_) to generate highly reactive hydroxyl radicals (•OH), and the accumulation of •OH leads to cell death [5]. Recently, chemodynamic therapy (CDT) for cancer treatment based on Fenton or Fenton-like reactions has garnered important attention [6–8] and copper is an outstanding CDT agent [9]. Moreover, the discovery and elucidation of cuproptosis [10], a distinct form of cell death characterized by copper caused aggregation of lipoylated tricarboxylic acid (TCA) cycle proteins, further boosted the development of cuproptosis-related drugs as novel anticancer strategies [11–13].

Despite the hopeful potential, the application of copper-based drugs faces several challenges: (a) As the excessive copper is harmful for normal tissues, a primary challenge lies in achieving precise delivery of sufficient copper to cancer cells while minimizing off-target leakage. (b) Excessive glutathione (GSH) and inadequate H_2_O_2_ impede the effect of copper-based drugs. GSH in tumor cells not only chelates with Cu^+^, inhibiting cuproptosis [10], but also reduces the •OH generated by Fenton-like reaction. Additionally, the intratumoral H_2_O_2_ is not adequate for Fenton-like reaction; thus, H_2_O_2_ supply by either direct delivery or peroxide materials is also necessary [14,15]. (c) Copper-based drug alone is insufficient to achieve satisfactory anticancer effect. Therefore, combination with other therapies is an attractive solution for enhanced efficacy.

Glucose oxidase (GOx) can act both as a H_2_O_2_ producer and a GSH scavenger. GOx oxidizes glucose to generate gluconic acid and H_2_O_2_, providing adequate H_2_O_2_ for Fenton-like reactions [16]. The increased H_2_O_2_ and its product •OH consume more GSH, which releases Cu^+^ to induce cuproptosis. Moreover, cancers exhibit a distinct metabolic profile compared to normal tissues, characterized by high consumption. Consequently, cutting off the supply of cancer fuels will effectively inhibit tumor growth, namely, starvation therapy [17,18]. Numerous strategies have been proposed [19–21], among which glucose starvation attracts the most attention, as glucose is the main energy source of cancer cells. GOx is a common agent for inducing glucose starvation [22,23]. Therefore, incorporating GOx to Cu-based drug endows them with synergistic therapy ability [24].

The expression of immune checkpoints facilitates tumor immune escape, among which programmed death ligand-1 (PD-L1) is the most extensively researched, and its inhibitors have already been employed in clinical treatment [25]. It is reported that intracellular copper up-regulates the PD-L1 expression on tumor cells [26] and cuproptosis is negatively correlated with tumor microenvironment (TME) score [27], both of which promote tumor immune escape. Therefore, combining copper-based drugs with anti-programmed death-1 antibody (αPD-1) could help address this issue and enhance their antitumor efficacy [28,29]. In addition, a series of nonapoptotic regulated cell death (RCD) like autophagy, ferroptosis, pyroptosis, and necroptosis were revealed to be associated with immunogenic cell death (ICD), which stimulates damage-associated molecular patterns (DAMPs) release and therefore triggers an adaptive immune response [30,31]. As a newly discovered RCD, cuproptosis was reported to be linked to ICD as well [28,32]. Besides, generation of reactive oxygen species (ROS), like •OH, is a crucial component of ICD, and antioxidants can diminish this process [33]. Thus, combining copper-based drugs with immune checkpoint blockade (ICB) may further enhance their antitumor effect.

**Fig. 1.**
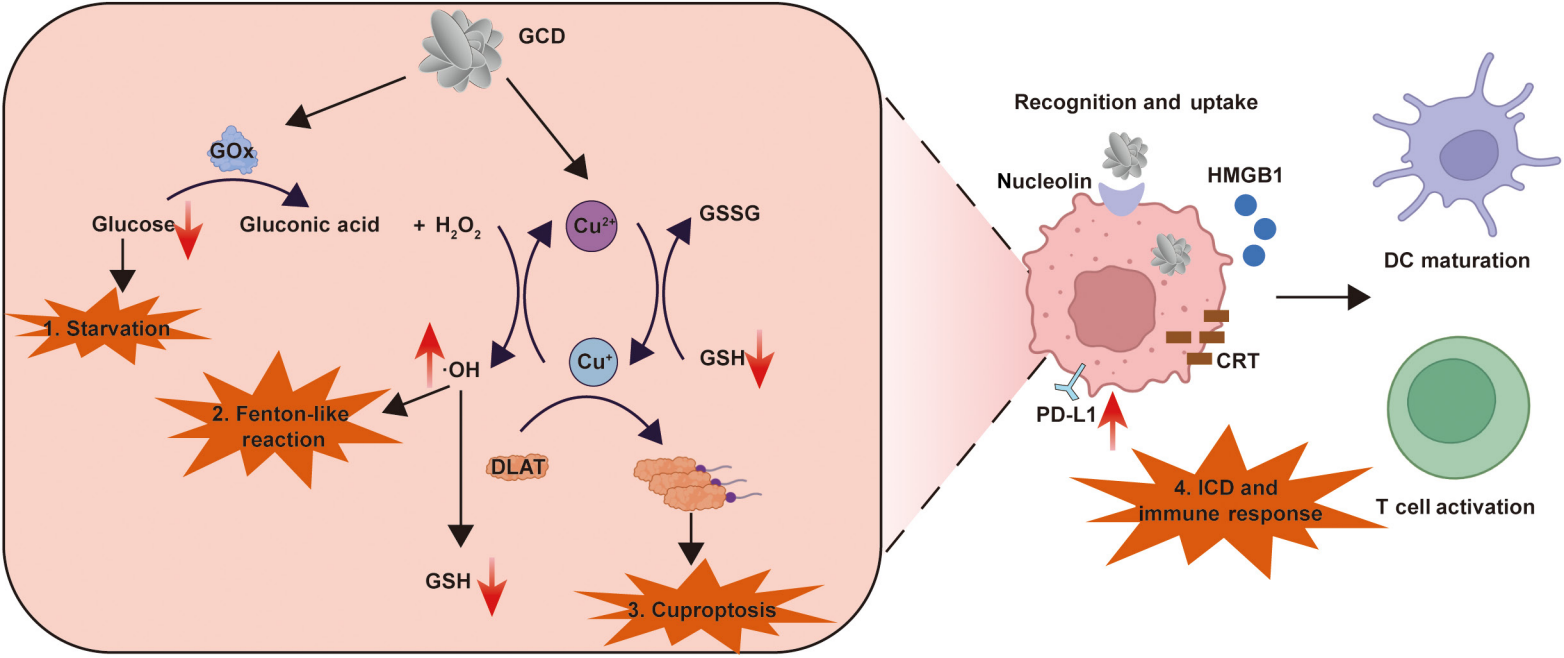
The synergistic anticancer effect of GCD. GOx not only causes glucose exhaustion (1. Starvation) but also provides adequate H_2_O_2_, which acts with Cu^+^ to trigger Fenton-like reaction (2. Fenton-like reaction). Besides, Cu^+^ induces oligomerization of DLAT, a core component of the pyruvate dehydrogenase complex, through disulfide stress-mediated protein misfolding, leading to cell death (3. Cuproptosis). In addition, copper ions up-regulates PD-L1 expression while GOx-generated ROS triggers ICD, remodeling the tumor immune microenvironment together (4. ICD and immune response).

Herein, we designed a multifunctional DNA-based nanodrug conjugate incorporating copper and GOx to exert antitumor roles. The AS1411 aptamer can bind to nucleolin, which is highly expressed on tumor cell membrane, enabling its targeted delivery to cancer cells [34]. To avoid leakage of GOx and copper to normal tissues, we designed a circular DNA template with sequence complementary to AS1411. Rolling circle amplification (RCA) generated a long single-stranded DNA (ssDNA) strand containing multiple repeated AS1411 aptamers. GOx and CuCl_2_ were then added to the system and biomineralization incorporated them into a flower-like structure [35] termed glucose oxidase-copper-DNA hybrid nanoflower (GCD). As shown in Fig. 1, AS1411 endows GCD with tumor-targeting characteristic. After being taken up by cancer cells, the acidic environment degraded the flower-like structure, releasing GOx and Cu^2+^ [36]. GOx not only exhausted intracellular glucose, but also provided more H_2_O_2_ for Fenton-like reaction (Eq. 1). Cu^+^, reduced from excessive exogenous Cu^2+^ by GSH, triggered cuproptosis (Eq. 2). Meanwhile, abundant •OH was generated via Fenton-like reaction (Eq. 3). Additionally, gluconic acid generated by glucose oxidation further decreased the pH, accelerating the degradation of GCD. The ROS generated through these cascading reactions not only directly induced cell death but also depleted excess GSH. ICD activated by cuproptosis and ROS promoted high mobility group box 1 (HMGB1) and calreticulin (CRT) release, dendritic cell (DC) maturation, and adaptive immune response, sequentially. In summary, we proposed a targeted synergistic cancer treatment strategy based on cuproptosis, glucose starvation, and immune modulation.

$$\text{Glucose}+O_{2}+H_{2}O\overset{\mathrm{GOx}}{\to }\;G\text{luconic acid}+H_{2}O_{2}$$ (1)

$$\mathrm{GSH}+{\mathrm{Cu}}^{2+}\to \text{GSSG}+{\mathrm{Cu}}^{+}$$ (2)

$${\mathrm{Cu}}^{+}+H_{2}O_{2}\to {\mathrm{OH}}^{-}+{\mathrm{Cu}}^{2+}+∙\mathrm{OH}$$ (3)

## Materials and Methods

### Materials and reagents

Cupric chloride dihydrate (CuCl_2_·2H_2_O, AR) and GOx were purchased from Macklin (Shanghai, China). Dimethyl sulfoxide (cell culture grade, >99.5%), 4′,6-Diamidino-2-phenylindole (DAPI) solution (>90%), 2′,7′-dichlorofluorescin diacetate (DCFH-DA), and LA Assay Kit (BC2230) were purchased from Solarbio Science & Technology Co. Ltd. (Beijing, China). φ29 DNA polymerase, T4 DNA ligase, and dNTPs were purchased from Sangon Biotech Co. Ltd. (Shanghai, China). DNA ladder and nucleic acid dye were purchased from Biosharp (Hefei, China). All DNA sequences used were synthesized and purified by Sangon Biotech Co. Ltd. Dulbecco’s modified Eagle’s medium (DMEM) culture medium was purchased from Gibco (Carlsbad, CA, USA). Roswell Park Memorial Institute (RPMI)-1640 culture medium and fetal bovine serum (FBS) were purchased from Viva Cell (Shanghai, China). Cell Counting Kit-8 (CCK-8) was purchased from Selleck (Shanghai, China). The Annexin V-FITC/PI Apoptosis Detection Kit was purchased from Vazyme (Jiangsu, China). Dihydrolipoamide S-acetyltransferase (DLAT) (13426-1-AP) lipoic acid synthetase (LIAS) (11577-1-AP) and HIF-1α (20960-1-AP) antibodies were from Proteintech (Wuhan, China). Glyceraldehyde -3-phosphate dehydrogenase (GAPDH) (A19056) and ferredoxin 1 (FDX1) (A20895) antibodies were from Abclonal (Wuhan, China). GOx (ab314545) antibody was purchased from Abcam (UK).

### Preparation of GCD

A linear padlock ssDNA with a sequence complementary to the AS1411 aptamer (red in Table S1) and a primer ssDNA (sequence shown in Table S1) were designed. Phosphorylated padlock DNA (100 ng) and 300 ng of primer were mixed in 1× T4 ligase buffer solution. The mixture was heated at 95 °C for 2 min, 55 °C for 5 min, and gradually cooled to 20 °C using a polymerase chain reaction thermal cycler. T4 DNA ligase (10 U) was added to the annealed solution, and the solution was incubated at 16 °C for 8 h and then 4 °C overnight to synthesize circular template. The solution was heated to 65 °C for 10 min to inactivate the T4 DNA ligase. For RCA, the circular DNA template (0.5 μM), dNTPs (1.25 mM), and 10 U φ29 DNA polymerase were added sequentially into 1× φ29 DNA buffer to a final volume of 40 μl. The reaction solution was kept at 37 °C for 4 h. After the RCA process, CuCl_2_ (10 mM), GOx (4 mg/ml), or both were added to incubate for another 48 h. The product was then washed with double-distilled H_2_O 3 times and stored at −20 °C.

### Characterization of GCD

DNA concentration of GCD was measured by a NanoDrop (Thermo Fisher Scientific, USA). The sizes of the circular template, long ssDNA achieved by RCA, and final product GCD were estimated by agarose gel electrophoresis on a 2.5% gel for 30 min (120 V). The gel was stained with nucleic acid dye and then imaged using the ChemiDoc XRS+ imaging system (Bio-Rad). The morphology of the products was examined by scanning electron microscopy (SEM, Noca NanoSEM 450). Particle diameters were determined by dynamic light scattering (DLS, Malvern Zetasizer Nano ZS90). X-ray photoelectron spectroscopy (XPS, Thermo Scientific K-Alpha) and inductively coupled plasma mass spectrometry (ICP-MS, PerkinElmer NexION 300X) were used to analyze element composition. We used Coomassie blue staining and Western blot (WB) to validate the existence of GOx in GCD. In brief, after sodium dodecyl sulfate-polyacrylamide gel electrophoresis (SDS-PAGE) electrophoresis, the gel was heated in ddH_2_O for 3 min. Then, Coomassie staining solution was added and incubated for 30 min. The gel was washed with ddH_2_O and photographed.

### Cell culture and cellular uptake

All cell lines were purchased from the American Type Culture Collection (Manassas, VA, USA). Human normal mammary epithelial cell line MCF-10A cells were cultured in specialized medium as per the manufacturer’s instructions. Mice breast cancer cell line 4T1, human breast cancer cell line MDA-MB-231, and human differentiated thyroid cancer cell line TPC1 cells were cultured in DMEM with 10% FBS. Human normal thyroid epithelial cell line Nthy-ori 3.1 cells were cultured in RPMI-1640 medium with 10% FBS. All cells were cultured in a 5% CO_2_ incubator under humidified 37 °C conditions.

For cellular uptake detection, a Cy5-labled probe was designed (Table S1) and mixed with GCD to produce GCD with red fluorescence. 4T1, MDA-MB-231, MCF-10A, TPC1, and Nthy-ori 3.1 were seeded in 6-well plates. After adherence, they were treated with 6 μg/ml GCD for 24 h. Then, the cells were fixed with 4% paraformaldehyde, stained with LysoTraker (green), and then observed using a confocal laser scanning microscope (Nikon, Japan).

### Cell toxicity assay

4T1, MDA-MB-231, and TPC1 cells were seeded in 96-well plates at a density of 8,000/well and cultured overnight for adherence. After different treatments (GCD, GCD with *N*-acetylcysteine (NAC) or UK5099, and compositions of GCD), the cell viability was examined using the CCK-8 assay. CCK-8 agent (10 μl) and 90 μl of RPMI-1640 medium were mixed and added to the cells for 1 h incubation. The absorbance at 450 nm was examined with a microplate analyzer (Thermo Fisher Scientific, USA).

### EdU assay

Cell proliferation ability was detected using an 5-ethynyl-2′-deoxyuridine (EdU) kit. Cells were seeded in 96-well plates at a density of 8,000/well. After adherence, 6 μg/ml GCD was added. After 24 h, cells were labeled with EdU, fixed, permeated, incubated with Click Reaction Buffer, and stained with Hoechst 33342 to show the nuclei following the manufacturer’s instructions. Images were captured using a fluorescence microscope (Nexcope, China). The ratio of EdU-positive cells was determined using ImageJ.

### Apoptosis assay

Terminal deoxynucleotidyl transferase dUTP nick-end labeling (TUNEL) and Annexin V/propidium iodide (PI) staining was used to evaluate cell apoptosis after incubation with GCD. Cells were seeded in 6-well plates and incubated with phosphate-buffered saline (PBS) or GCD at a concentration of 6 μg/ml for 24 h. For TUNEL staining, cells were fixed with 4% paraformaldehyde and stained using the TUNEL kit and then with DAPI. The images were collected using a confocal laser scanning microscope (CLSM). For Annexin V/PI staining, the cells were incubated with GCD, GCD with NAC or UK5099, and compositions of GCD. After digesting and resuspension in 100 μl of binding buffer, 5 μl of Annexin V-fluorescein isothiocyanate (FITC) and PI were added and incubated at room temperature for 10 min in the dark. Flow cytometry (LSRFortessa X-20; BD) was used to analyze apoptosis rates. The data analysis was performed using FlowJo software.

### Western blot

4T1 cells were seeded in 6-well plates and treated with GCD as described above. Cells were washed with PBS and then lysed with RIPA buffer containing protease inhibitors for 10 min. Total cellular protein was separated using SDS-PAGE, transferred to nitrocellulose membranes, and then incubated with primary antibodies at 4 °C overnight and secondary antibodies at room temperature for 1 h. Tris-buffered saline Tween (TBST) was used to wash the nitrocellulose membranes. The proteins of interest were visualized and analyzed with ECL and the ChemiDoc XRS+ imaging system.

### Migration ability

Cellular migration ability was evaluated using wound healing and transwell assays. For wound healing, cells were seeded in 6-well plates. After GCD treatment and culturing to full confluence, a straight line was scratched on the cell monolayer using a 1-ml sterile pipette tip and washed with PBS. Cells were cultured in 1% FBS medium afterward and images were captured by a microscope (Nexcope, China) immediately and every 24 h until fuse. For transwell, 2 × 10^5^ cells in serum-free medium were seeded in the upper chamber. Medium with 20% serum was placed in the lower chamber. After culturing for 24 h, migrating cells were fixed with 4% paraformaldehyde for 15 min, then stained with 1% crystal violet for 30 min, and the images were captured after washing.

### H_2_O_2_ detection

A hydrogen peroxide assay kit (Nanjing Jiancheng, A064-1-1) was used to detect H_2_O_2_ production according to its manufacturer’s instructions. The H_2_O_2_ concentrations of (a) 500 μM H_2_O_2_, (b) 500 μM H_2_O_2_ + 6 μg/ml GCD, and (c) 500 μM H_2_O_2_ + 6 μg/ml GCD + 5 mM glucose were detected every 3 min.

### GSH detection

Cells were seeded in 6-well plates and treated with GCD as described above. After incubation for 24 h, cells were washed 3 times with PBS and lysed. The concentration of GSH was measured according to the manufacturer’s instructions (Nanjing Jiancheng, A006-2-1).

### ROS generation

A total of 5,000 cells were seeded in 24-well plates and treated with GCD as described above. After incubation for 24 h, the culture medium was removed and the cells were washed 3 times with PBS. DCFH-DA (10 μM) mixed in serum-free culture medium was used to incubate the cells for 30 min. After the incubation, cells were washed 3 times with PBS and fresh culture medium was added. A fluorescence microscope was used to capture the images.

### Qualification of glucose consumption

To calculate the consumption of glucose, the medium before and after cell culture was collected and the glucose concentration was detected by a Glucose Kit (Nanjing Jiancheng, A154-1-1) according to the manufacturer’s instructions. The consumption was indicated by the difference of glucose concentration before and after cell culture.

### Qualification of LA

The concentration of intracellular lactic acid (LA) was detected according to the manufacturer’s instructions of LA Assay Kit (Solarbio, BC2230). In brief, extract agent was added to the cells after different treatments. After sonication and centrifugation, the supernatant containing LA was added to 96-well plates and incubated with working fluid for 30 min. The absorbance at 570 nm was measured and used to calculate LA concentration.

### Energy metabolism detection

The oxygen consumption rate (OCR) and extracellular acidification rate (ECAR) were measured by a 24-well Seahorse XFe Extracellular Flux Analyzer (Seahorse XFe24, Agilent) according to the manufacturer’s instructions. In brief, the 4T1 cells were treated with PBS or GCD for 24 h and then seeded in a Seahorse XFe24 cell culture microplate and cultured overnight. After that, the culture medium was replaced with specifical medium and cultured for another 1 h. During the test, glucose and oligomycin were injected sequentially for ECAR, and oligomycin, fluorocarbonyl cyanide phenylhydrazone (FCCP), and rotenone plus antimycin A (Rot/AA) were injected sequentially for OCR.

### Immunofluorescence assay

Cells were seeded in confocal dishes, treated with GCD, and fixed as described above. Cells were blocked using 1% bovine serum albumin and then incubated with primary antibodies and Cy3-labeled or Dylight488-labeled secondary antibodies. Images were captured using a confocal microscope.

### Enzyme-linked immunosorbent assays

All enzyme-linked immunosorbent assay (ELISA) kits were purchased from Elabscience, and the assays were performed according to the ELISA kit manufacturer’s instruction. For HMGB1 detection, the culture medium supernatant of GCD-treated 4T1 cells was used. For interleukin-6 (IL-6) and IL-12, the medium of cocultured DCs was used. For tumor necrosis factor-α (TNF-α) and interferon-γ (IFN-γ), the tumors of orthotopic breast cancer model were used.

### DC maturation

Bone marrow-derived dendritic cells (BMDCs) were isolated from hind limbs of 6-week female Balb/c mice. The cells were cultured in RPMI-1640 with 10% FBS, 1% penicillin/streptomycin, GM-CSF (20 ng/ml), and IL-4 (5 ng/ml) in a 5% CO_2_ incubator under humidified 37 °C conditions. Half-medium was changed every 2 days. Semi-suspended cells and loosely adherent cells were collected on day 8.

4T1 cells were seeded in 6-well plates and treated with GCD for 24 h after adherence. Afterward, the harvested immature BMDCs were added to the cells and cocultured for an additional 24 h. Then, the BMDCs were stained with anti-CD11c-APC, anti-CD80-FITC, and anti-CD86-PE-Cy5.5. Flow cytometry was used to analyze the ratio of mature DCs (CD80^+^, CD86^+^). The supernatant of culture medium was collected for ELISA of IL-6 and IL-12. The ELISA process followed the manufacturer’s instruction.

### Hemolysis experiment

Mouse blood was collected and mixed with saline at a ratio of 1:9 and then centrifuged at room temperature at 10,000 *g* for 5 min. The supernatant was discarded and the precipitant was washed with saline 3 times to obtain the red blood cells (RBCs). The RBCs were then resuspended in 10 ml of saline. RBC suspension (200 μl) was incubated with 800 μl of H_2_O for positive control, 800 μl of PBS for negative control, and 800 μl of saline containing 3 mg of GCD for the experimental group at 37 °C for 30 min and then centrifuged at 2,000 rpm for 5 min. The absorbance of the supernatant was measured at 577 nm.

### Orthotopic breast cancer model

All animal experiments were performed according to the animal use and care regulation and the animal management rules of the Ministry of Health of the People’s republic of China ([2022] IACUC Number: 4101). The Balb/c mice were bought from GemPharmatech (Jiangsu, China). Mice were housed in an specific pathogen-free environment and were free to eat and drink. A total of 2 × 10^5^ 4T1 cells were suspended in 100 μl of PBS and implanted into mammary fat pads of female Balb/c mice. The tumor size was measured every 3 days. Tumor volume was calculated according to the following formula: volume = (short axis)^2^ × (long axis)/2.

### In vivo evaluation of the GCD antitumor effect

After 5 days of inoculation, mice bearing orthotopic breast tumors were randomly divided into 4 groups (*n* = 5), including (i) PBS, (ii) GCD, (iii) αPD-1, and (iv) GCD+αPD-1. GCD (7.5 g/kg/day) was injected intraperitoneally on days 1, 4, 7, and 10. αPD-1 (3 mg/kg/day) was injected intraperitoneally on days 2, 5, 8, and 11. After 12 days of treatment, mice were sacrificed. Livers and kidneys were harvested for hematoxylin and eosin (H&E) staining to evaluate the bio-safety. Tumors were harvested for H&E and immunohistochemistry (IHC) staining. For flow cytometry analysis, tumor tissues were cut into small pieces, digested in DMEM medium with 1 mg/ml collagenase IV, and 0.02 mg/ml DNaseI at 37 °C for 1 h, and stained with anti-CD45-APC-H7, anti-CD3-BV421, anti-CD4-BV605, and anti-CD8-PE-CF594. The lymph nodes were placed in a dish, ground into single cells, and stained with anti-CD11c-APC, anti-CD80-FITC, and anti-CD86-PE-Cy5.5.

### Statistical analysis

All statistical analyses were performed with GraphPad Prism 9.0 software (San Diego, CA). The significance of the difference was evaluated by grouped 2-tailed Student’s *t* tests for 2-group data and 2-way analysis of variance for multigroup data. It was considered statistically significant if the *P* value was less than 0.05 (*), 0.01 (**), 0.001 (***), or 0.0001 (****).

## Results

### Synthesis and characterization of GCD

**Fig. 2.**
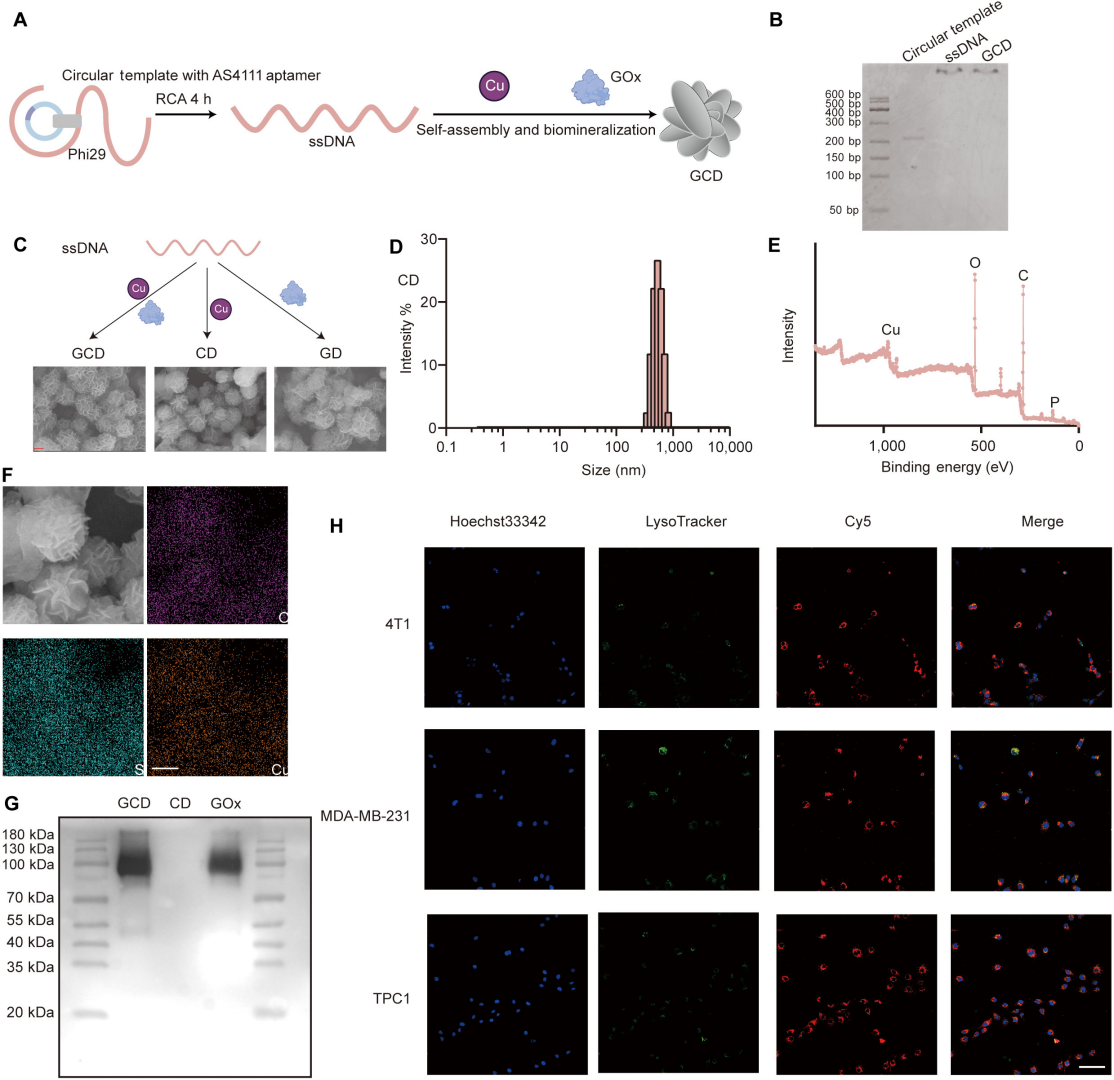
Synthesis and characterization of GCD. (A) Schematic illustration of the synthesis of GCD. (B) Native-PAGE showing the long ssDNA of GCD. (C) SEM images of GCD, GD, and CD. Scale bar: 500 nm. (D) Size distribution of GCD by DLS. (E) Full range XPS of GCD showing the element composition. (F) EDS of O, S, and Cu in GCD; scale bar: 500 nm. (G) WB of GCD, GD, and GOx. (H) Representative CLSM images of 4T1, MDA-MB-231, and TPC1 cells after incubation with Cy5-GCD (red) for 24 h. Nuclei were stained with Hoechst33342 (blue); scale bar: 100 μm.

A linear ssDNA containing sequence complementary to the AS1411 aptamer (Table S1) was designed to prepare the circular template for RCA, which is a flexible process to produce multifunctional DNA materials. After 4 h of RCA, CuCl_2_ and GOx were added, and the mixture underwent an additional 48 h of biomineralization to form the DNA nanoplatform incorporating GOx and Cu^2+^ (GCD) (Fig. 2A). Native-PAGE electrophoresis (Fig. 2B) verified the synthesis of long ssDNA produced by RCA (lane 2), which exhibited minimal migration in the gel. The subsequent addition of CuCl_2_ and GOx did not disrupt this structure (lane 3). SEM (Fig. 2C) confirmed the flower-like morphology of GD (added with GOx only), CD (added with CuCl_2_ only), and GCD (added with GOx and CuCl_2_). DLS indicated an average diameter for GCD of approximately 542.0 ± 108.7 nm (Fig. 2D). To better understand the composition of GCD, XPS (Fig. 2E) and energy-dispersive spectroscopy (EDS) (Fig. 2F) were employed, demonstrating the uniform distribution of oxygen (O), copper (Cu), and sulfur (S) in the flower-like structure of GCD. Inductively coupled plasma mass spectrometry (ICP-MS) determined the concentrations of S and Cu in GCD to be 75 and 90 mg/L, respectively. Since S is a characteristic element of proteins, the aforementioned results confirmed the successful incorporation of both CuCl₂ and GOx into the GCD nanostructure. WB (Fig. 2G) and Coomassie blue staining (Fig. S1) provided further evidence for the presence of GOx. Lanes 1 (GCD) and 3 (GOx) exhibited identical bands, whereas no corresponding band was detected in lane 2 (GD), indicating the exhibition of GOx in GCD.

To evaluate the tumor-targeting ability of GCD, we labeled GCD with Cy5 and incubated mice breast cancer cell line 4T1, human breast cancer cell line MDA-MB-231, human normal mammary epithelial cell line MCF-10A, human differentiated thyroid cancer cell line TPC1, and human normal thyroid epithelial cell line Nthy-ori 3.1 with Cy5-GCD (red) and then LysoTracker (green). Images captured by CLSM (Fig. 2H and Fig. S2A and B) showed intense red fluorescence in 4T1, MDA-MB-231, and TPC1 cells, indicating efficient cellular uptake in these cells. In contrast, minimal fluorescence was observed in MCF-10A and Nthy-ori 3.1, suggesting that the uptake of GCD has tumor specificity. However, when the complementary sequence of AS1411 was replaced with poly-A, the uptake of tumor cells dramatically decreased (Fig. S2C).

These results successfully validated the preparation, structural characteristics, and tumor cell-specific uptake of GCD, providing a foundation for further investigation of its biological functions.

### GCD suppresses proliferation and migration of cancer cells in vitro

**Fig. 3.**
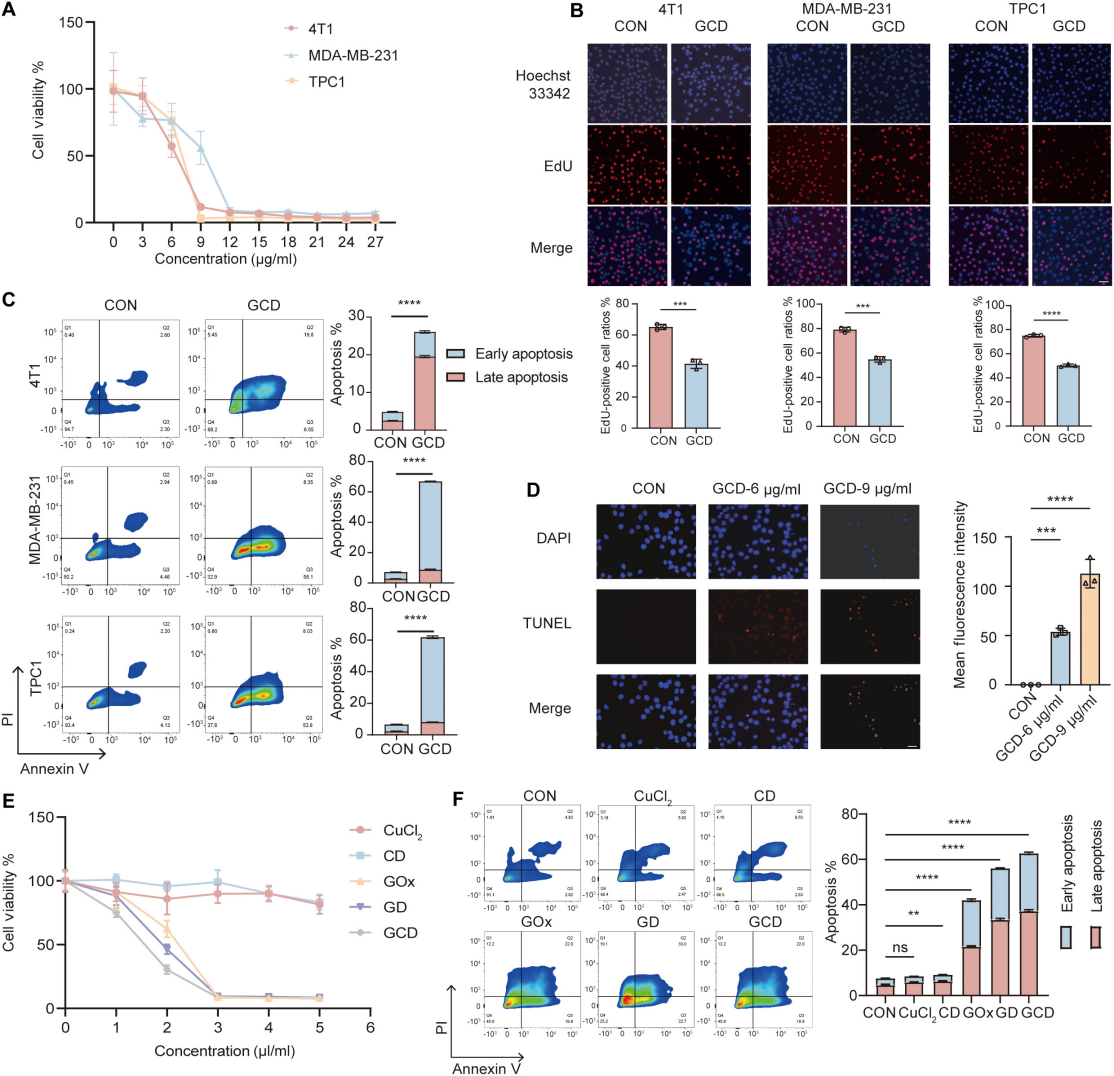
GCD-induced apoptosis of cancer cells in vitro*.* (A) Relative cell viability of 4T1, MDA-MB-231, and TPC1 cells measured by CCK-8 after incubation with GCD for 24 h. (B) EDU staining of 4T1, MDA-MB-231, and TPC1 cells in the absence or presence of GCD for 24 h; scale bar: 40 μm, *n* = 3. (C) Representative flow cytometry plots of 4T1, MDA-MB-231, and TPC1 cells after incubation with GCD for 24 h (left) and their calculated apoptosis rates (right), *n* = 3. (D) TUNEL staining of 4T1 cells after incubation with 6 or 9 μg/ml GCD for 24 h; scale bar: 20 μm, *n* = 3. (E) Relative cell viability following treatment with equivalent concentrations of Cu^2+^ or GOx, *n* = 5. (F) Representative flow cytometry plots following treatment with equivalent concentrations of Cu^2+^ or GOx (left) and their calculated apoptosis rates (right) , *n* = 3. ns: *P* ≥ 0.05, **P<0.01, ***P<0.001, ****P<0.0001.

To investigate GCD’s antitumor effect in vitro in pan-cancers, we treated mice breast cancer cell line 4T1, human breast cancer cell line MDA-MB-231, and human differentiated thyroid cancer cell line TPC1 cells with it. These tumor cells were derived from multiple species and diverse organ systems, demonstrating the broad-spectrum antitumor efficacy of GCD. CCK-8 assay (Fig. 3A) and EdU assay (Fig. 3B) revealed that GCD significantly inhibited cell viability and DNA replication in these cancer cells. Cellular viability decreased dramatically at the concentration of 9 μg/ml in 4T1 and TPC1 cells, and 12 μg/ml in MDA-MB-231 cells. Annexin V/PI staining demonstrated that GCD increased apoptosis ratio in all cell lines (Fig. 3C). Both early and late apoptotic rates showed at least a 2-fold increase. TUNEL staining (Fig. 3D) further verified GCD-induced cell damage. When treated with 6 μg/ml GCD, the red fluorescence of 4T1 cells was enhanced, while when treated with 9 μg/ml GCD, the red fluorescence was much brighter and the morphology of nuclei shown by DAPI staining was changed, indicating that GCD induced cell apoptosis and the effect increased with the concentration of GCD. Together, these results demonstrated that GCD significantly induced apoptosis. To evaluate the contribution of the individual components of GCD, we treated 4T1 cells with CuCl_2_, CD, GOx, GD, and GCD (with equivalent concentrations of Cu^2+^ or GOx). CuCl_2_ and CD showed minimal cytotoxicity, while GCD exhibited the strongest effect. Dose-equivalent cytotoxicity comparisons (Fig. 3E and Table S2) revealed that, at 2 μg/ml GCD-equivalent copper, neither CuCl₂ nor CD reduced viability beyond 20%. With GCD-equivalent GOx (2 μg/ml), free GOx decreased viability to 62% (*P* < 0.001) and GD further reduced viability to 46% (*P* < 0.0001). Strikingly, the full GCD complex achieved maximal inhibition (30% viability, *P* < 0.0001), demonstrating synergistic toxicity. Quantification of apoptosis (Fig. 3F) demonstrated similar results. CuCl₂ treatment induced no significant increase in apoptotic cells (*P* > 0.05), and CD complex caused a marginal increase of 2% (*P* = 0.0044). Free GOx and GD complex increased apoptosis by 34% (*P* < 0.0001) and 48% (*P* < 0.0001), respectively. Notably, the GCD complex elicited a substantial increase of 55% (*P* < 0.0001).

Subsequently, the impact of GCD on cell migration was assessed. Figure S3A showed that wound healing rate was obviously slowed down in the GCD group at both 24 h and 48 h. Transwell assay revealed a more than 50% decrease in the migration ratio following GCD treatment (Fig. S3B). Taken together, these results suggested that GCD treatment impeded cell proliferation and migration, and enhanced cell apoptosis, indicating strong antitumor efficacy of GCD in vitro.

### GCD enhances ROS generation, cuproptosis, and metabolism alterations

**Fig. 4.**
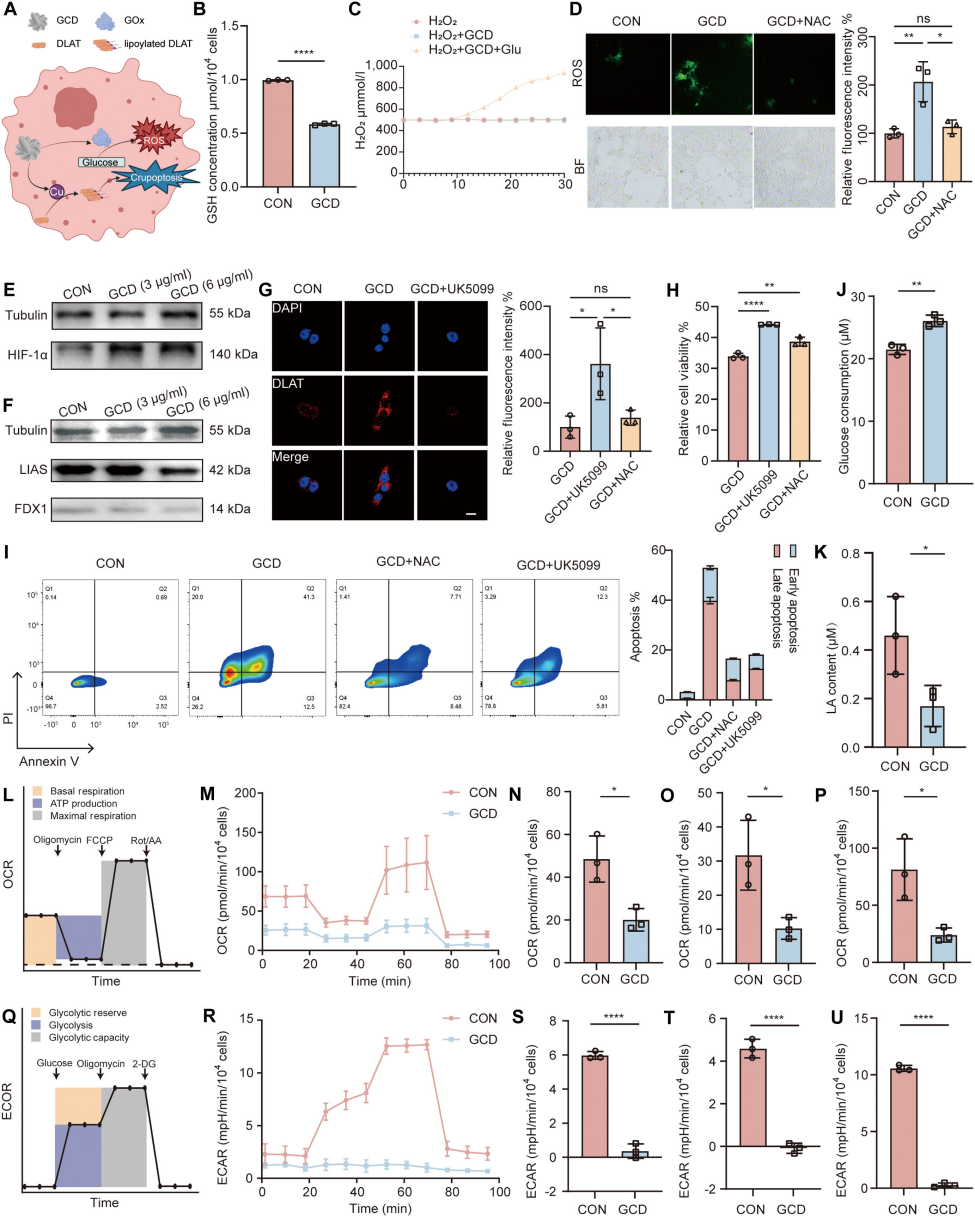
In vitro evaluation of ROS release and cuproptosis induced by GCD. (A) Proposed scheme of GCD inducing ROS generation and cuproptosis in cells. (B) GSH concentration after GCD treatment, *n* = 3. (C) H_2_O_2_ production by GCD or GCD plus glucose. (D) Generation of ROS detected by DCFH-DA; scale bar: 40 μm, *n* = 3. (E) Levels of HIF-1α after incubation with 3 and 6 μg/ml GCD. (F) Levels of cuproptosis markers after incubation with GCD. (G) DLAT staining (red) of 4T1 cells after incubation with GCD and rescue with UK5099. Nuclei were stained with DAPI (blue); scale bar: 10 μm, *n* = 3. (H) Relative cell viability after different treatments, *n* = 3. (I) Cell apoptosis after different treatments, *n* = 3. (J) Glucose consumption detected by the glucose concentration of culture medium, *n* = 3. (K) Cellular LA concentration of 4T1 cells treated with PBS or GCD, *n* = 3. (L) Profile of the OCR during the glycolysis stress test. (M) Real-time OCR of 4T1 cells after treatment with GCD. (N) Basal respiration, (O) ATP-linked respiration, and (P) maximal respiration of 4T1 cells with different treatment, *n* = 3. (Q) Profile of the ECAR during the Mito stress test. (R) Real-time ECAR of 4T1 cells after treatment with GCD. (S) Glycolysis, (T) glycolytic reverse, and (U) glycolytic capacity of 4T1 cells with different treatment, *n* = 3. ns: *P* ≥ 0.05, *P<0.05, **P<0.01, ****P<0.0001.

Encouraged by the promising in vitro antitumor results, we further investigated the mechanism underlying the GCD’s multifunctional antitumor capability. Under neutral physiological conditions, the nanoflower structure remained stable, minimizing the premature release of GOx and Cu^2+^. In an acid TME, however, the nanoflower degraded due to the decomposition of magnesium pyrophosphate at acidic pH [36], releasing GOx and Cu^2+^. GOx catalyzed glucose oxidation with the by-product of H_2_O_2_ and gluconic acid, which simultaneously deprived tumor cells of glucose and provided H_2_O_2_ for Fenton-like reactions. Cu^2+^ aided Fenton-like reactions through 2 primary pathways: (a) participation in Fenton-like reactions to generate cytotoxic •OH and (b) depletion of intracellular GSH via oxidating it to oxidized glutathione (GSSG) [37], preventing •OH scavenging. Meanwhile, overload of copper in tumor cells initiated cuproptosis, an RCD pathway mediated by lipoylated protein aggregation in the TCA cycle [10]. Together, GCD simultaneously induced oxidative stress with self-supplementation of H_2_O_2_ and cuproptosis, and the 2 processes mutually reinforced each other (Fig. 4A).

Experimental validation confirmed these mechanisms. First, GSH detection was performed, and it was found that GCD treatment significantly reduced the GSH concentration in 4T1 cells (Fig. 4B), indicating the GSH depletion role of GOx in GCD. As depicted in Fig. 4C, the addition of GCD and glucose led to H_2_O_2_ production, while GCD alone did not result in significant changes in H_2_O_2_ concentration. This result confirmed that GCD oxidizes glucose to generate H_2_O_2_ in vitro. We employed DCFH-DA, a fluorescent probe whose intensity correlates with ROS levels, to further evaluate the ROS production in cells. Following GCD treatment, a marked increase in green fluorescence intensity was observed, reaching 2-fold of the control group (*P* = 0.0056); however, the fluorescence was significantly attenuated upon the addition of the antioxidant NAC (Fig. 4D), which inhibited ROS production [38]. Meanwhile, oxidative stress marker HIF-1α [39] level significantly increased after treatment with GCD (1.499 vs. 1.049, *P* = 0.025) (Fig. 4E). To evaluate cuproptosis induced by GCD, LIAS and FDX1 expressions were detected by WB (Fig. 4F) and DLAT aggregation was detected by CLSM (Fig. 4G). Expression levels of LIAS (0.3585 vs. 1.0680, *P* < 0.0001) and FDX1 (0.3972 vs. 1.045, *P* < 0.0001) were significantly decreased after 24-h incubation with 6 μg/ml GCD. Obvious red fluorescence scatters were observed in the GCD-treated group, suggesting massive DLAT aggregation while the DLAT aggregation in the control group was negligible. Besides, cotreatment with UK5099, a potent inhibitor of the mitochondrial pyruvate carrier known to suppress cuproptosis [10], attenuated the fluorescence intensity. Crucially, parallel experiments in human normal mammary epithelial cell line MCF-10A and human normal thyroid epithelial cell line Nthy-ori 3.1 revealed no significant changes after the 6 μg/ml GCD treatment. ROS detection assays revealed that while GCD induced a 2-fold increase in fluorescence intensity (*P* = 0.0056) in tumor cells, it only caused marginal changes in normal cells: 124.3% in MCF-10A (*P* = 0.3934) and 126.9% in Nthy-ori 3.1 (*P* = 0.1194) (Fig. S4A). Expression of HIF1-α (0.9853 vs. 1.007, *P* = 0.4704 in MCF-10A; 1.1610 vs. 0.9783, *P* = 0.0588 in Nthy-ori 3.1), LIAS (1.0230 vs. 1.0610, *P* = 0.6192 in MCF-10A; 1.0700 vs. 0.9308, *P* = 0.1692 in Nthy-ori 3.1), and FDX1 (1.0530 vs. 1.0970, *P* = 0.8107 in MCF-10A; 1.0330 vs. 1.1270, *P* = 0.4458 in Nthy-ori 3.1) showed no significant difference as well (Fig. S4B). This differential response confirms GCD’s tumor-targeting specificity.

Following the verification of ROS generation and cuproptosis, we subsequently assessed whether inhibitors targeting the 2 progresses were able to rescue the cells. The CCK-8 assay showed that cell viability was partially restored upon the addition of either UK5099 or NAC (Fig. 4H). Similarly, apoptosis was effectively inhibited by treatment with UK5099 and NAC (Fig. 4I). Besides, hypoxia and cuproptosis markers evaluated by WB were rescued as well (Fig. S5). Taken together, these results suggested that GCD induces cell death through synergistic ROS generation and cuproptosis.

Given that cuproptosis is mitochondrial related and GOx can cut off the primary energy supply of tumors, we hypothesized that GCD induces metabolism alterations in tumor cells. As glycolysis is the predominant energy supply pathway for tumors and GOx can oxidize its substrate glucose, we first measured glucose consumption in 4T1 cells treated with PBS or GCD. The results showed that the glucose consumption increased from 21.50 to 26.08 μM/day after GCD was added (Fig. 4J), due to the oxidation ability of GOx. Consequently, the utilizable glucose for tumor cells was significantly reduced. Afterward, we assessed the effect of GCD on the concentration of LA, the product of glycolysis. It is not surprising to find that GCD treatment reduced intracellular LA concentration to less than half of that in untreated controls (Fig. 4K). To further clarify the effect of GCD on energy metabolism, Seahorse analyzer was used to examine the OCR (Fig. 4L), reflecting oxidative phosphorylation (OXPHOS), and ECAR (Fig. 4Q), reflecting glycolysis, respectively. As for OCR (Fig. 4M), basal respiration (Fig. 4N), adenosine triphosphate (ATP)-linked respiration (Fig. 4O), and maximal respiration (Fig. 4P) were substantially inhibited following GCD incubation. Meanwhile, as for ECAR (Fig. 4R), glycolysis (Fig. 4S), glycolytic reserve (Fig. 4T), and glycolytic capacity (figure 4U) were decreased in the GCD-treated group. Collectively, these results demonstrated that the main energy supply pathways, namely, glycolysis and mitochondrial OXPHOS, were both hindered by GCD, indicating its role in blocking energy supply.

### GCD promotes ICD and DC maturation

**Fig. 5.**
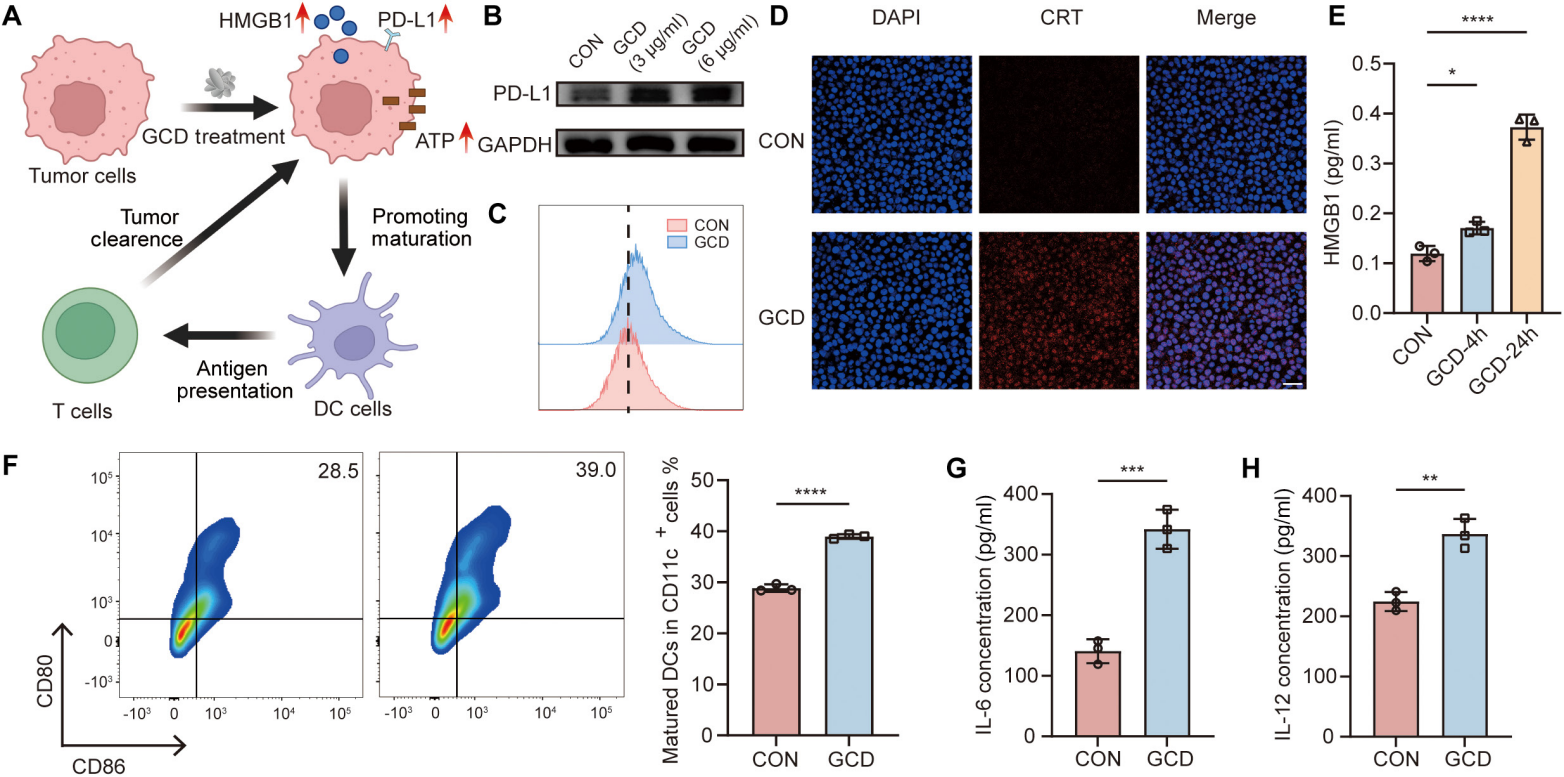
ICD and DC maturation induced by GCD. (A) Proposed scheme of GCD inducing ICD and DC maturation. PD-L1 expression level after treatment with GCD of different concentration (3 and 6 μg/ml) detected by WB (B) and flow cytometry (C). (D) CRT staining (red) of 4T1 cells after incubation with GCD; nuclei were stained with DAPI (blue); scale bar: 40 μm. (E) HMBG1 release after incubation with GCD, *n* = 3. (F) Representative flow cytometry plots of DC maturation stimulated by GCD-incubated 4T1 cells (left) and percentage of mature DCs (CD80^+^CD86^+^) of total CD11c^+^ cells (right) , *n* = 3. IL-6 (G) and IL-12 (H) release by BMDC after coculture with GCD-treated 4T1 cells, *n* = 3. *P<0.05,**P<0.01, ***P<0.001, ****P<0.0001.

Copper overload has been reported to up-regulate PD-L1 expression on the surface of tumor cells [26], which plays a significant role in immune escape. To validate the effect of GCD on PD-L1 expression, WB (Fig. 5B) and flow cytometry (Fig. 5C and Fig. S6) assays were executed. The mean expression of PD-L1 in cells treated with 3 and 6 μg/ml GCD was 1.8-fold (*P* = 0.04) and 2.1-fold (*P* = 0.01) of untreated controls, implying that combining GCD with αPD-1 therapy may enhance antitumor efficacy. Furthermore, it is reported that cuproptosis may be able to trigger the release of DAMPs such as HMGB1 and CRT, which are the hallmarks of ICD [40,41]. These DAMPs also recruit immune cell infiltration and activate APCs (such as DCs) to evoke immune response [42,43]. We therefore hypothesized that GCD stimulates HMGB1 release and CRT surface expression, promotes DCs maturation, and ultimately enhances CD8^+^ T cell infiltration and tumor clearance. To further evaluate the immunoregulation role of GCD, we first detected exposure of CRT by immunofluorescence. In the GCD-treated group, an obvious increase of red fluorescence on the cell periphery was observed compared to the control group, indicating elevated CRT exposure (Fig. 5D). HMGB1 release detected by ELISA yielded consistent results. The concentration of HMGB1 in the culture medium supernatant grew with the extension of incubation time, and 24-h treatment increased HMGB1 levels to over 3-fold of the control group (Fig. 5E). Inspired by the remarkable increase of ICD markers, we next detected its DC maturation promoting role in vitro. Immature BMDCs from Balb/c mice were cocultured with GCD-treated 4T1 cells for 24 h. As evaluated by flow cytometry, the expression of CD80 and CD86, as classical costimulatory molecules and mature DC markers, were significantly improved in the GCD-treated group (Fig. 5F), indicating enhanced mature DCs. The proportion of mature DCs was 1.35-fold of the control group. In addition, concentrations of the mature DC-related cytokines IL-6 and IL-12 in the supernatant were 2.43- and 1.50-fold of the control group, respectively (Fig. 5G and H). Above all, GCD treatment triggered PD-L1 up-regulation, ICD, and DC maturation (Fig. 5A), which motivated us to further evaluate the therapeutic effect of combining GCD with ICB treatment in mouse models.

### In vivo anti-tumor effect of GCD

**Fig. 6.**
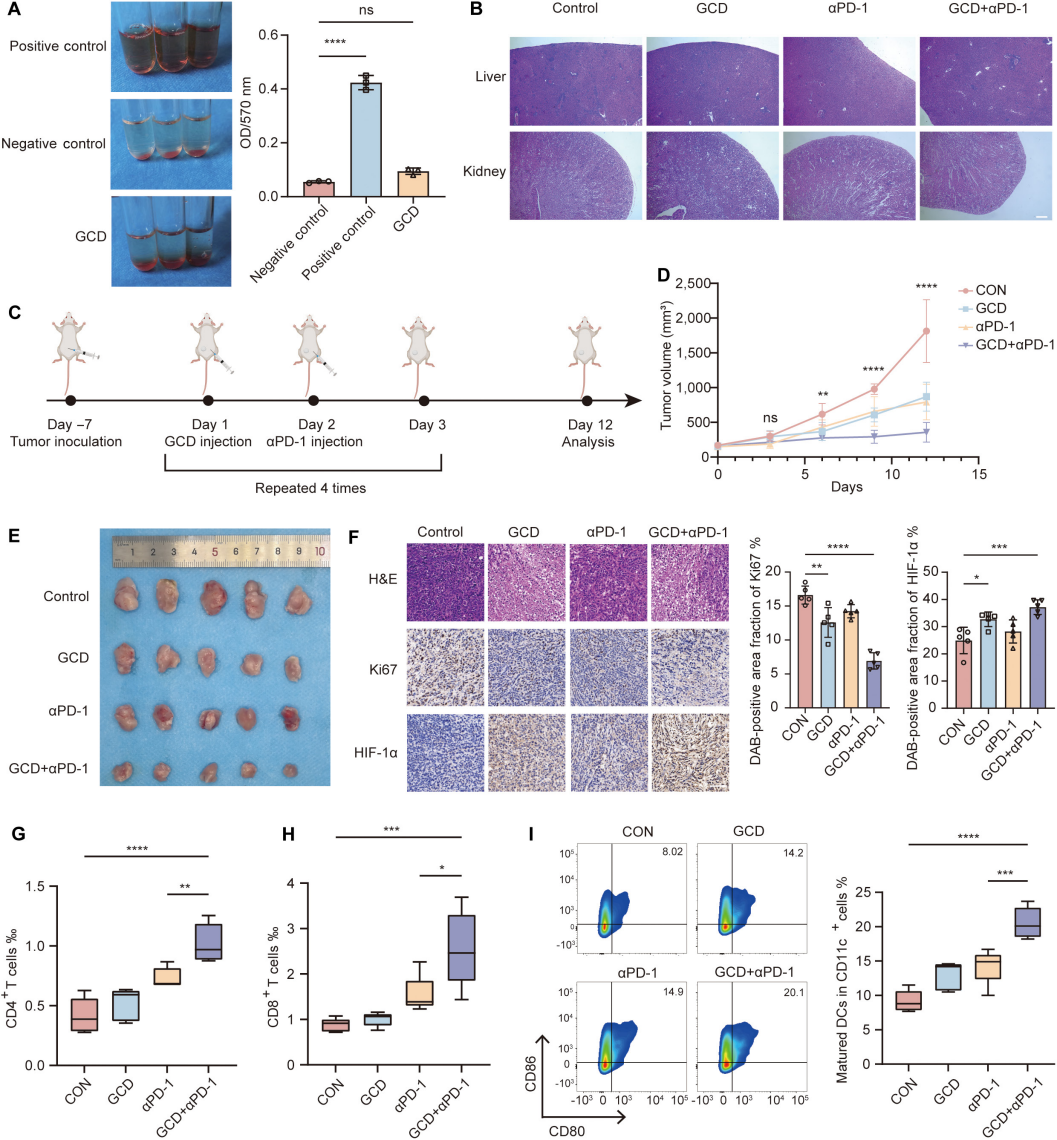
In vivo evaluation of antitumor effects of GCD. (A) Hemolysis analysis of GCD, *n* = 3. (B) H&E staining of liver and kidney after injection of GCD for 12 days; scale bar: 100 μm. (C) Scheme of animal experiments. (D and E) Tumor-bearing mice received different treatments and the tumor volume was measured every 3 days. (F) H&E, Ki-67, and HIF-1α staining of tumors with different treatments; scale bar: 100 μm, *n* = 5. (G to I) Flow cytometry analysis of percentage of CD4^+^ T cells (G) and CD8^+^ T cells (H) of total cells in tumor and mature DCs (I) (CD80^+^CD86^+^) of total CD11c^+^ cells in lymph nodes, *n* = 5. ns: *P* ≥ 0.05, *P<0.05, **P<0.01, ***P<0.001, ****P<0.0001.

Prior to in vivo application, hemolysis experiment was performed to assess safety. As shown in Fig. 6A, there was no obvious hemolysis in the GCD-treated group. In addition, H&E staining revealed no histopathological abnormality in GCD-treated mice (Fig. 6B). These results indicated that GCD exhibits satisfying biocompatibility.

We designed in vivo experiments as illustrated in Fig. 6C to investigate the tumor inhibition effect of GCD monotherapy and its combination with αPD-1. In brief, 4T1 cells were orthotopically inoculated into the mammary fat pads of Balb/c mice. After 7 days, GCD was injected every 3 days, followed by αPD-1 injection the next day. Tumor sizes were measured every 3 days and tumor growth curves were plotted (Fig. 6D). The mice were sacrificed after 4 cycles of treatment. There was a significant decrease in tumor volume in the GCD/αPD-1 monotherapy group, and the combination therapy group showed a dramatic further suppression of tumor growth (Fig. 6E). To understand the mechanism underlying the antitumor effect of GCD in vivo, IHC was used to detect the expression levels of Ki-67 and HIF-1α (Fig. 6F). The combination group exhibited significantly higher expression of HIF-1α but lower expression of Ki-67, which is consistent with previous findings. Additionally, H&E staining revealed noticeably increased necrosis in the combination group. Corroborating with previous results, these findings indicated that GCD is a promising therapeutic agent for tumors.

Based on the in vitro results, we explored the immune regulation role of GCD in vivo. To verify the immune cell recruiting effect, T cells in tumor tissues were analyzed by flow cytometry (Fig. 6G and H). T cell infiltration was notably increased in the combination therapy group, reaching almost twice that of the control group. ELISA assays showed that GCD significantly elevated cytokine secretion related to T cell function like IFN-γ and TNF-α (Fig. S7). Additionally, the proportion of mature DCs in collected lymph nodes was increased as well (Fig. 6I), which facilitated antigen presentation and subsequent immune-mediated tumor clearance. These results indicated that GCD possesses potent antitumor efficacy and synergizes with immunotherapy.

## Discussion

This study introduces a multifunctional DNA nanohydrogel platform (GCD) for the codelivery of copper and GOx, killing tumor cells and remodeling the TME through combined CDT and enhanced antitumor immunity. We successfully confirmed that GCD comprises a well-defined, robust DNA hydrogel formed from a long ssDNA scaffold. Tandem-repeat AS1411 aptamers, generated via RCA, equip it with specific tumor-targeting capability. GCD dissociates within the acidic TME, enabling the controlled release of Cu^2+^ and GOx, which act synergistically to induce oxidative stress and cuproptosis. Furthermore, GCD disrupts cancer cell energy supply and therefore inhibits proliferation and metastasis. Besides, it induces ICD and maturation of DCs to potentiate ICB efficacy. Preclinical in vivo studies demonstrated that GCD exhibits potent tumor suppression, robust immune activation, and minimal side effects. In summary, this DNA nanohydrogel-based combination therapy, integrating glucose deprivation, CDT, cuproptosis, and immune remodeling, addresses the multifaceted requirements of anticancer strategies and holds significant promise as a platform candidate for clinical translation.

As a critical component of this study, the bio-safety and tumor-targeting ability of the GCD nanogel was comprehensively evaluated through both in vitro and in vivo assays. GCD exhibited nonsignificant toxicity comparable to the control group and minimal hemolysis. This demonstrates the favorable biocompatibility and safety profile of GCD, which are essential for assessing the in vivo potential and clinical applications. The superior targeting capability of GCD enables it to deliver the cargo precisely and reduce leakage to normal tissues. Nucleolin is a multifunctional protein localized to the nucleolus and plasma membrane of malignant cells, offering a targeting pathway for tumor-selective delivery platforms [44]. The AS1411 aptamer targeting nucleolin exhibits high binding affinity toward tumor cells and has emerged as a significant tool for the detection and treatment of malignancies [45]. In this work, the formation of tandem repeat AS1411 units via RCA equipped GCD with tumor targeting ability, which was confirmed by confocal fluorescence microscopy. In brief, fluorescently labeled DNA probe was used to label GCD via base complementary pairing. After the incubation, significant fluorescence signal accumulation within cancer cells was observed, with minimal signal in normal cells.

Although the mechanism remains incompletely understood, the recently identified cuproptosis has been highlighted as a distinct strategy for adjunctive cancer therapy. In addition, copper-based Fenton-like reactions have advantages such as rapid kinetics and operation at a favorable pH range [46,47], rendering them particularly suitable for developing CDT nanomaterials. Consequently, copper-based materials hold significant promise in antitumor research. However, insufficient H₂O₂ levels and the hypoxic microenvironment in tumors constrain the efficiency of CDT. To address this limitation, prior studies have explored the codelivery of copper ions and H₂O₂ [48,49]. Nevertheless, cuproptosis induction and CDT show insufficient potency for effective antitumor roles. In this study, we integrated copper delivery with GOx-mediated self-supplying H₂O₂ strategy. This approach not only enhanced CDT and cuproptosis efficacy but also disrupted tumor energy metabolism, achieving a synergistic integration of CDT and glucose deprivation-based starvation therapy.

Mounting evidence supports the existence of cascade ICD [50], wherein dying tumor cells release antigens that recruit DCs for antigen presentation. Mature DCs subsequently activate T cells, ultimately stimulating antitumor immunity [42]. Herein, we demonstrated GCD-induced ICD in cancer cells through in vitro detection of HMGB1 release and CRT surface exposure. DC coculture assays confirmed a potent maturation-promoting effect, which was further substantiated by in vivo models showing GCD-enhanced DC maturation and increased T cell infiltration. Consistent with recent advances [28,29], we established the synergistic antitumor efficacy of GCD combined with αPD-1 therapy in vivo. Therefore, combining cuproptosis/CDT/starvation therapy/TME reprogramming induced by copper-based nanomaterials with conventional immunotherapy represents a promising anticancer strategy.

However, this study has certain limitations. First, the mechanism underlying how copper regulates PD-L1 expression remains unclear. While several studies reported synergistic effects between copper-based agents and αPD-1 therapy [28,29], few have comprehensively explored the underlying molecular and signaling pathways. Future studies are warranted to elucidate this process. Besides, the targeting ability of AS1411 relies on the expression of nucleolin. In this study, its targeting specificity was only assessed in a limited panel of cell lines, and its universality needs further research in multiple cancer cell lines of diverse origins.

This copper-based synergistic tumor-targeted strategy might hold promise for tumor treatment. Since the DNA nanoflower is easily programmable, the addition of variable motifs will assign it with more functions such as pH response and more precise targeting. Besides, the platform offers the potential to load additional therapeutic agents to further boost antitumor efficacy. In summary, our study provides an in-depth exploration of combining cuproptosis with other therapeutic modalities, such as metabolic intervention and immune modulation. We anticipate that this work will inspire the design and application of novel copper-based synergistic anticancer drugs.

## Data Availability

All data used related to the current study are available from the paper, the Supplementary Materials, or the corresponding authors on reasonable request.
