# Supplementary material for: Nucleolin-Targeted DNA Nanoflowers Enable Multimodal Synergistic Cancer Therapy
Source: Biomater Res. 2025 Sep 23;29:0254. doi: 10.34133/bmr.0254 (PMC12454938; doi:10.34133/bmr.0254)
Supplement: Supplementary 1 — Tables S1 and S2 Figs. S1 to S7 [file bmr.0254.f1.doc]

**Supporting Information**

**Nucleolin targeted DNA Nanoflowers Enables multimodal** **Synergistic Cancer therapy**

Anwen Ren ^1^, Huan Liu ^1^, Zimei Tang ^1^, Qingyi Hu ^1, #^, Tao Huang ^1, #^

TABLE OF CONTENTS

**1. Supplementary tables.**

Table S1

Table S2

**2. Supplementary figures.**

Figure S1

Figure S2

Figure S3

Figure S4

Figure S5

Figure S6

Figure S7

1. **Supplementary Table.**

**Table S1. DNA sequence used in the study.**

| Name | Sequence (5’ – 3’) |
| --- | --- |
| Primer | CTGACGCCCGTTGCAGCTACC |
| Padlock ssDNA-AS1411 | ACGGGCGTCAGTGCTTCCGAAATGCTTGCCACCACCACCAACACCACCACCACCGTAGTTACGAAGGCGAGGTAGCTGCA |
| Padlock ssDNA-nontarget | ACGGGCGTCAGTGCTTCCGAAATGCTTGAAAAAAAAAAAAAAAAAAAAAAAAAAGTAGTTACGAAGGCGAGGTAGCTGCA |
| Probe | CGGGCGTCAGTGCTTCCGAA-Cy5 |

Sequence complementary to AS1411 aptamer was shown in red.

**Table S2. Statistical analysis of cytotoxicity assay of individual components of GCD.**

| Treatment | Group | Difference with 95% CI | Summary | P Value |
| --- | --- | --- | --- | --- |
| CuCl_2_ | 0 vs. 1 | 0.08644(-0.02247 ～ 0.1954) | ns | 0.2027 |
|  | 0 vs. 2 | 0.1406(0.03168 ～ 0.2495) | ** | 0.0038 |
|  | 0 vs. 3 | 0.101(-0.007896 ～ 0.2099) | ns | 0.0855 |
|  | 0 vs. 4 | 0.09938(-0.009529 ～ 0.2083) | ns | 0.095 |
|  | 0 vs. 5 | 0.1851(0.07616 ～ 0.2940) | **** | <0.0001 |
|  |  |  |  |  |
| CD | 0 vs. 1 | -0.009879(-0.1188 ～ 0.09903) | ns | 0.9998 |
|  | 0 vs. 2 | 0.04218(-0.06673 ～ 0.1511) | ns | 0.8715 |
|  | 0 vs. 3 | 0.009566(-0.09935 ～ 0.1185) | ns | 0.9999 |
|  | 0 vs. 4 | 0.09958(-0.009337 ～ 0.2085) | ns | 0.0939 |
|  | 0 vs. 5 | 0.1675(0.05856 ～ 0.2764) | *** | 0.0003 |
|  |  |  |  |  |
| GOx | 0 vs. 1 | 0.08727(-0.02165 ～ 0.1962) | ns | 0.194 |
|  | 0 vs. 2 | 0.3767(0.2678 ～ 0.4856) | **** | <0.0001 |
|  | 0 vs. 3 | 0.9105(0.8016 ～ 1.019) | **** | <0.0001 |
|  | 0 vs. 4 | 0.9199(0.8110 ～ 1.029) | **** | <0.0001 |
|  | 0 vs. 5 | 0.9186(0.8096 ～ 1.027) | **** | <0.0001 |
|  |  |  |  |  |
| GD | 0 vs. 1 | 0.1184(0.009479 ～ 0.2273) | * | 0.0247 |
|  | 0 vs. 2 | 0.5359(0.4270 ～ 0.6448) | **** | <0.0001 |
|  | 0 vs. 3 | 0.9049(0.7959 ～ 1.014) | **** | <0.0001 |
|  | 0 vs. 4 | 0.9084(0.7995 ～ 1.017) | **** | <0.0001 |
|  | 0 vs. 5 | 0.9139(0.8050 ～ 1.023) | **** | <0.0001 |
|  |  |  |  |  |
| GCD | 0 vs. 1 | 0.2484(0.1395 ～ 0.3573) | **** | <0.0001 |
|  | 0 vs. 2 | 0.6958(0.5868 ～ 0.8047) | **** | <0.0001 |
|  | 0 vs. 3 | 0.9147(0.8057 ～ 1.024) | **** | <0.0001 |
|  | 0 vs. 4 | 0.9187(0.8097 ～ 1.028) | **** | <0.0001 |
|  | 0 vs. 5 | 0.9216(0.8127 ～ 1.030) | **** | <0.0001 |

1. **Supplementary Figures.**


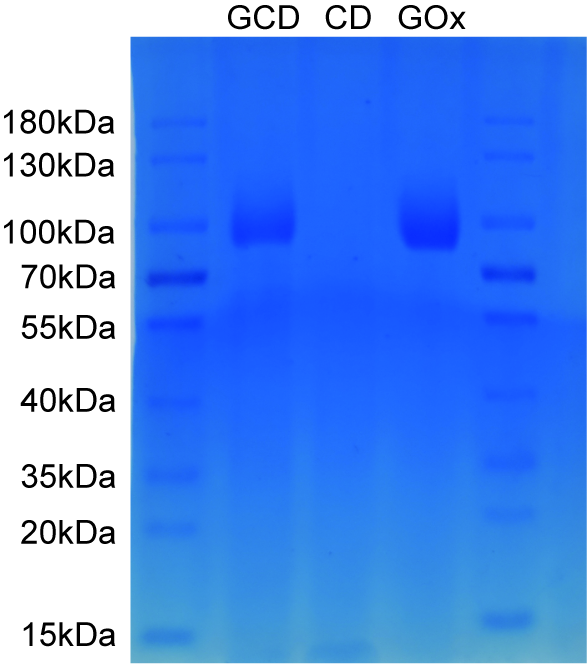


**Figure S1.** Coomassie blue staining of GCD, GD, and GOx.


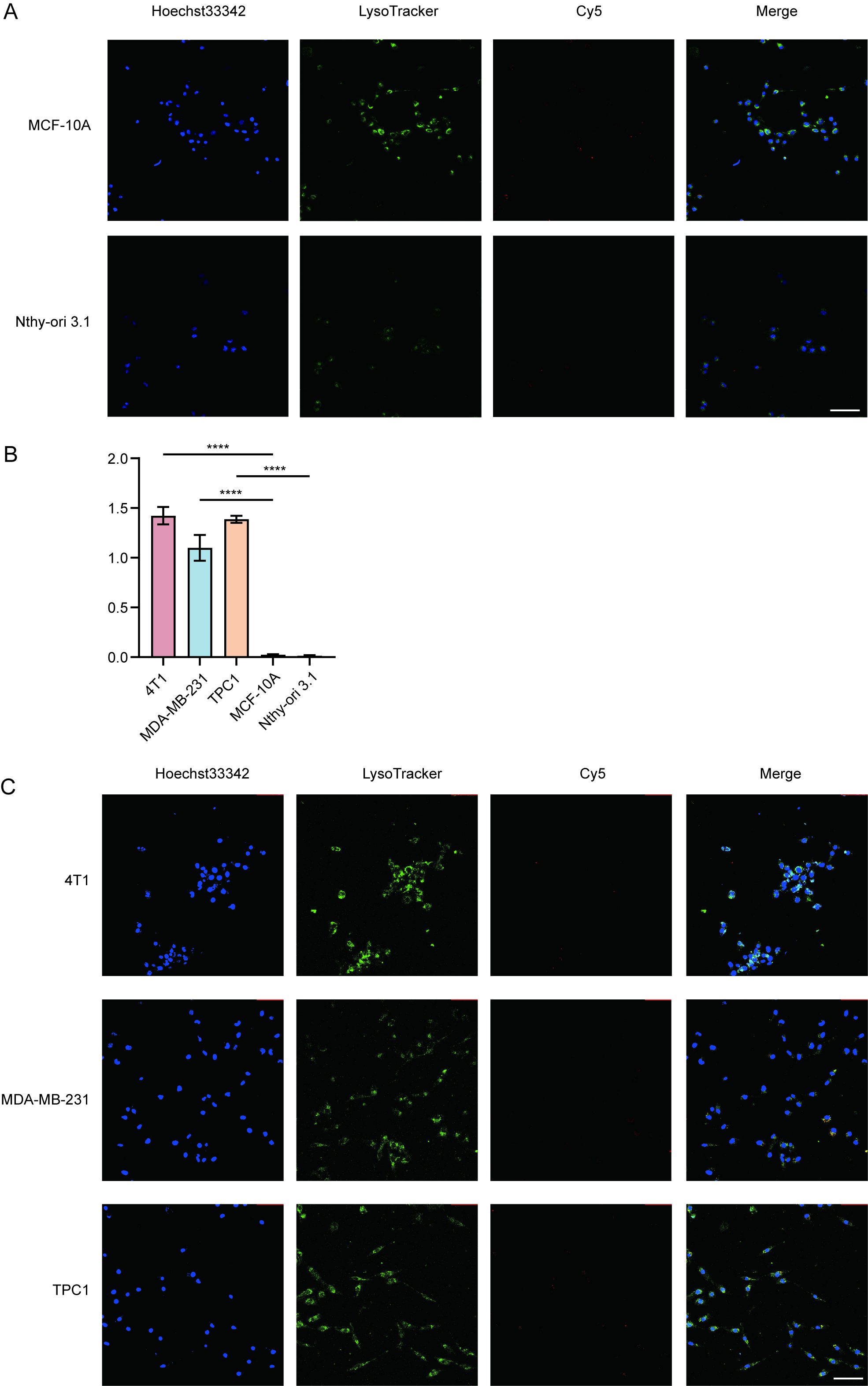


**Figure S2.** Cellular uptake of GCD. (A) Representative CLSM images of MCF-10A and Nthy-ori 3.1 cells after incubation with Cy3-GCD (red) for 24h, and nuclei were stained with Hoechst33342 (blue), scale bar: 100 μm. (B) Bar graph of normalized fluorescence intensity (GCD-Cy5/DAPI ratio) of 4T1, MDA-MB-231, TPC1, MCF-10A, and Nthy-ori 3.1 cells. (C) Representative CLSM images of 4T1, MDA-MB-231, and TPC1 cells after incubation with Cy5-GCD-non (red) for 24h, and nuclei were stained with Hoechst33342 (blue), scale bar: 100 μm.

**
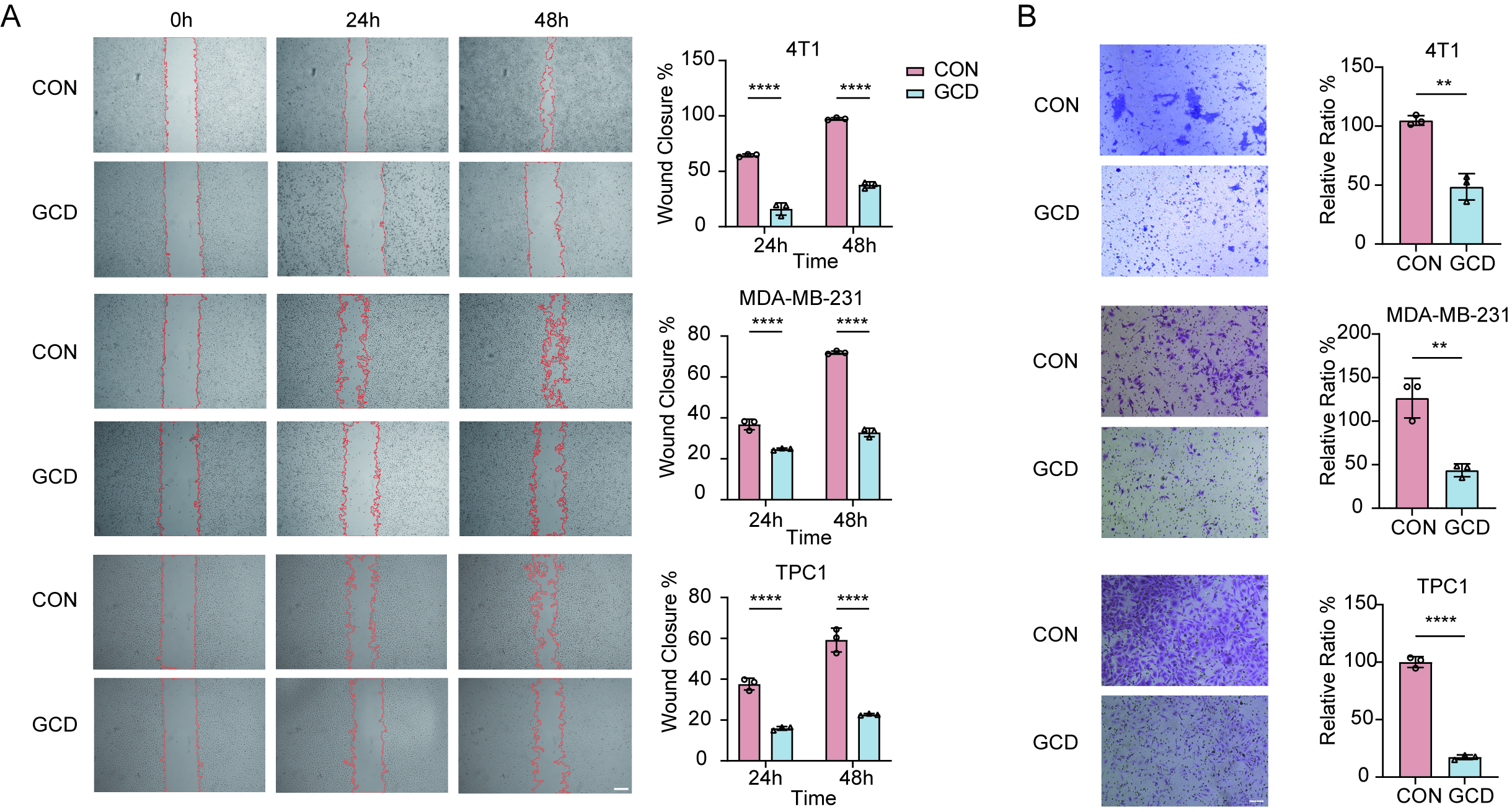
**

**Figure S3.** Migration inhibition roles of GCD. (A) Wound healing assay revealing the migration ability of 4T1, MDA-MB-231, and TPC1 cells after incubation with GCD for 24 hours, scale bar: 100 μm, n=3. (B) Transwell assay revealing the migration ability after treatment with GCD, scale bar: 40 μm, n=3. **: *P*＜0.01, ***: *P*＜0.001, ****: *P*＜0.0001.

**
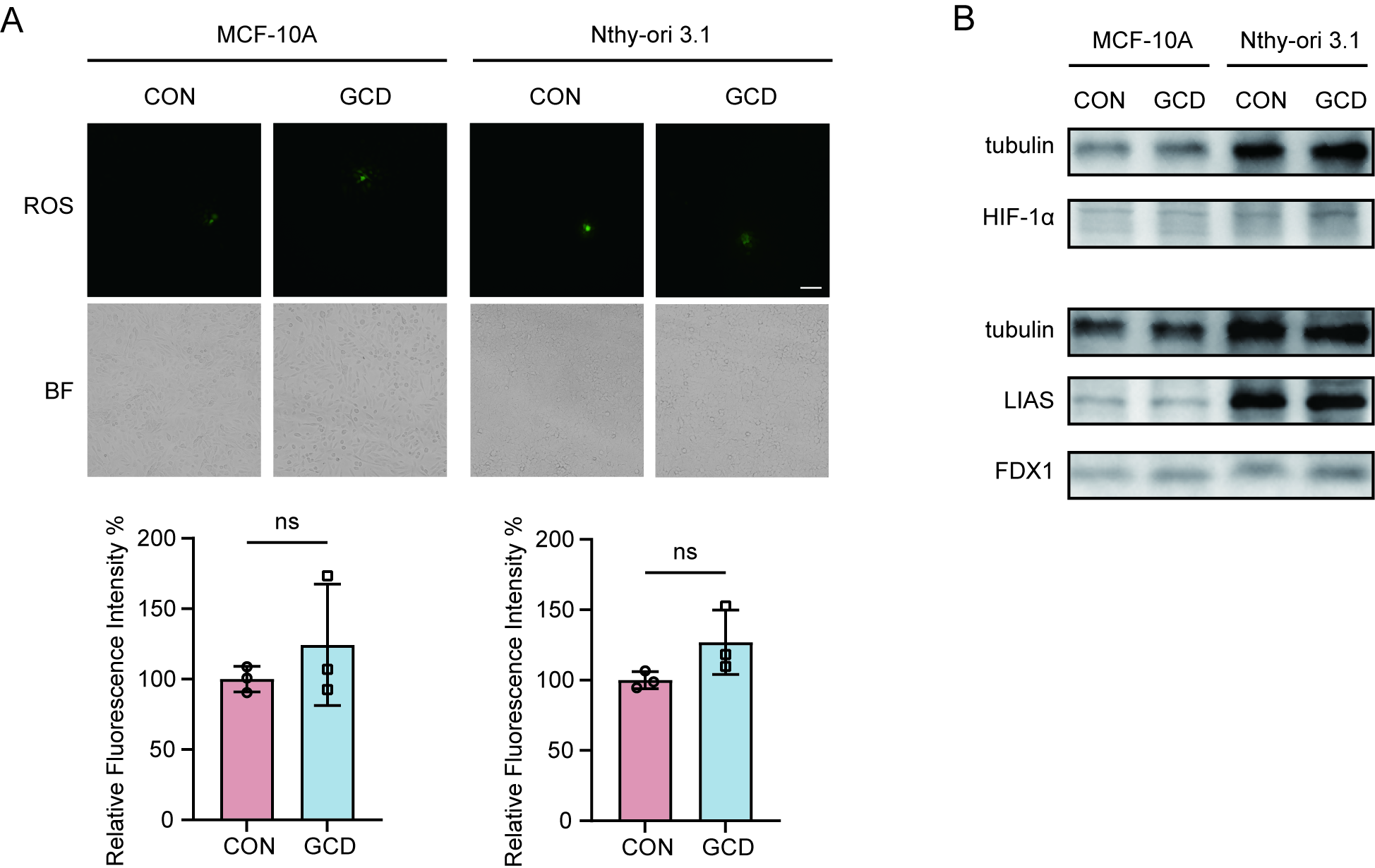
**

**Figure S4.** GCD did not induce obvious ROS generation and cuproptosis in normal cells. (A) Generation of ROS detected by DCFH-DA, scale bar: 40 μm, n=3. (B) Levels of HIF-1α and cuproptosis markers after incubation with 6 μg/mL GCD. ns: *P*≥0.5.


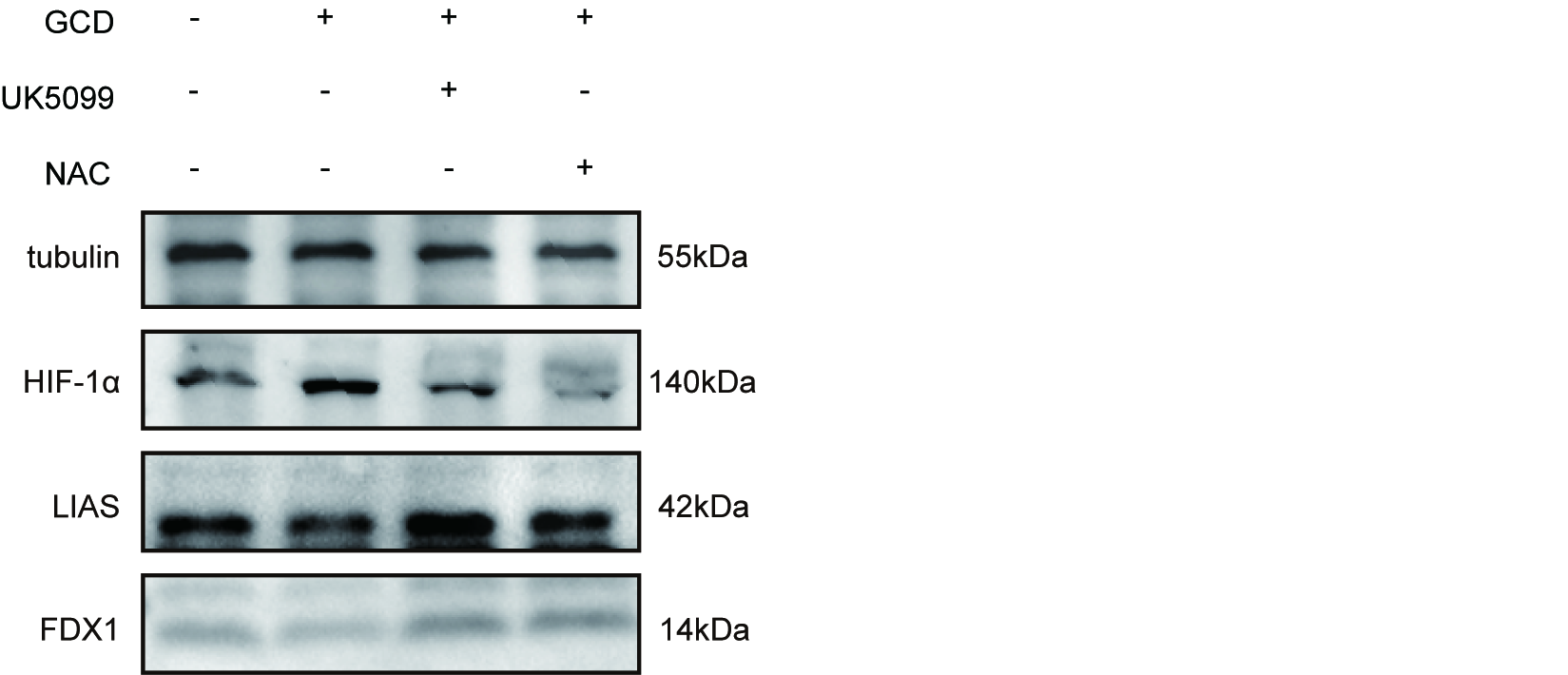


**Figure S5.** Levels of HIF-1α and cuproptosis markers after different treatments.


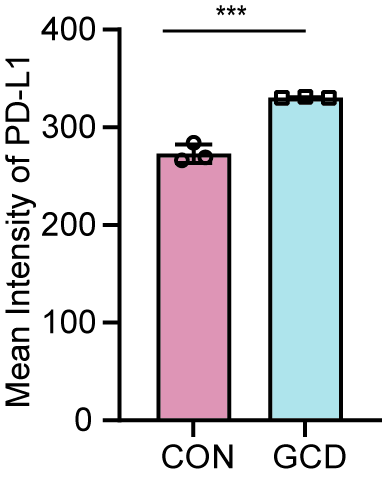


**Figure S6.** Statistic graph of flow cytometry results of PD-L1 expression.


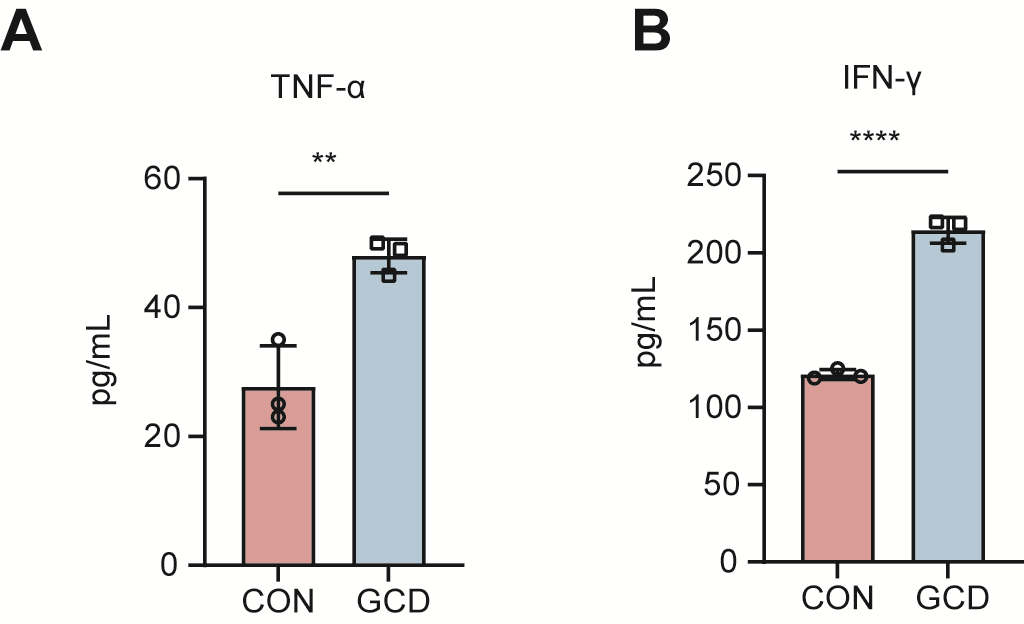


**Figure S7.** TNF-α (A) and IFN-γ (B) concentration in tumor tissues after GCD treatment.
